# Development of a Visual Rapid Assay for Novel Goose Astrovirus Detection Based on RT-MIRA -*Pf*Ago

**DOI:** 10.3390/bios16080433

**Published:** 2026-08-08

**Authors:** Dongdong Yin, Xinjun Chen, Zhixing Cheng, Yu Liu, Yin Dai, Xuehuai Shen, Xiaocheng Pan

**Affiliations:** 1 Anhui Provincial Key Laboratory of Livestock and Poultry Product Safety, Institute of Animal Husbandry and Veterinary Science, Anhui Academy of Agricultural Sciences, Livestock and Poultry Epidemic Diseases Research Center of Anhui Province, Hefei 230031, China; dalin2080@aaas.org.cn (Y.D.); shenxh@aaas.org.cn (X.S.); 2 Yebio Bioengineering Co., Ltd of Qingdao, Qingdao 266113, China; chenxinjun@yebio.com (X.C.); chengzhixing@yebio.com (Z.C.); liuyu344@yebio.com (Y.L.)

**Keywords:** goose astrovirus, multienzyme isothermal rapid amplification, *Pf*Ago, visual detection, rapid diagnosis

## Abstract

Goose astrovirus genotype 2 (GAstV-2) is an important pathogen associated with gosling gout, and rapid detection is useful for early diagnosis and field surveillance. In this study, a visual assay for GAstV-2 detection was developed by combining one-step reverse transcription multienzyme isothermal rapid amplification (MIRA) with the nucleic acid cleavage activity of *Pyrococcus furiosus* Argonaute (*Pf*Ago). MIRA primers and specific guide DNAs were designed based on a conserved region of the GAstV-2 *ORF1b* gene, and the *Pf*Ago reaction conditions were optimized. The optimal reaction contained 1.0 μM gDNA, 0.6 μM *Pf*Ago, and 1.0 mM MnCl_2_. Using recombinant pUC57-ORF1b plasmid DNA as the template, the lowest detectable plasmid concentration under the tested conditions was 1.0 × 10^0^ copies/μL. In the specificity assay, only GAstV-2 produced a positive signal, with no cross-reaction observed with GAstV-1, Tembusu virus, H9-subtype avian influenza virus, goose circovirus, fowl adenovirus serotype 4, or goose parvovirus. The assay was further tested with 23 clinical samples suspected of GAstV-2 infection. In a preliminary evaluation of 23 clinical samples, the RT-MIRA-*Pf*Ago results were concordant with those obtained by conventional RT-PCR and RT-qPCR. Overall, the RT-MIRA-*Pf*Ago assay provided sensitive and specific GAstV detection within a short time, without requiring programmed thermal cycling or an expensive real-time PCR instrument for routine endpoint detection. This method may be useful for GAstV-2 detection in basic laboratories and field settings.

## 1. Introduction

Goose astrovirus (GAstV) is a non-enveloped, positive-sense single-stranded RNA virus in the genus *Avastrovirus*, family *Astroviridae*. Since 2016, GAstV-associated gosling gout has occurred in several major goose-producing provinces in China. The disease is mainly characterized by urate deposition in visceral organs and joints. In some outbreaks, morbidity has reached 80% and mortality up to 50%, causing considerable losses to the goose industry [1,2,3,4]. Based on whole-genome sequences and capsid protein amino acid sequences, circulating GAstV strains are generally classified into two genotypes, GAstV-1 and GAstV-2. The newly emerged virus initially described as novel goose astrovirus is currently generally classified as GAstV-2, which has been predominantly associated with recent outbreaks of gosling gout [5,6]. Infections have also been reported in Cherry Valley ducks, Muscovy ducks, and chickens, indicating that the host range of GAstV-2 may extend beyond geese [7,8,9]. These observations highlight the need for diagnostic tools that can detect GAstV-2 rapidly and reliably, particularly during the early stage of infection.

Current laboratory diagnosis of GAstV mainly depends on virus isolation and molecular assays. Although virus isolation is considered a reference method, it is slow, labor-intensive, and poorly suited to routine rapid diagnosis. Reverse transcription polymerase chain reaction (RT-PCR) and real-time quantitative PCR (RT-qPCR) are widely used because of their sensitivity and specificity, but both require thermal cyclers, laboratory infrastructure, and trained personnel [10,11,12,13,14]. This limits their use on small farms or in field settings [15]. In addition to conventional RT-PCR and RT-qPCR, digital PCR provides sensitive absolute nucleic-acid quantification but requires specialized equipment. Isothermal amplification platforms include LAMP and recombinase-based methods such as recombinase polymerase amplification, enzymatic recombinase amplification, and multienzyme isothermal rapid amplification (MIRA) [16,17]. These amplification methods can be combined with fluorescence, lateral-flow, or colorimetric readouts. More recently, CRISPR/Cas- and prokaryotic Argonaute-assisted detection, microfluidic platforms, and integrated point-of-care systems have been developed to improve sequence specificity and portability [4,15]. Among these methods, LAMP reduces dependence on thermal cycling, but nonspecific amplification remains a potential source of false-positive results. MIRA is an isothermal amplification method that works at 25 °C–42 °C. With the combined action of recombinase, single-stranded DNA-binding protein, and strand-displacing DNA polymerase, target nucleic acids can be amplified within 10–30 min. However, when MIRA is used alone, primer dimers or nonspecific amplification products may produce false-positive signals, especially in complex clinical samples [18,19].

The Argonaute protein from *Pyrococcus furiosus* (*Pf*Ago) is a DNA-guided endonuclease. Under high-temperature conditions, *Pf*Ago cleaves complementary target sequences guided by 5′-phosphorylated single-stranded DNA [20]. Unlike CRISPR/Cas-based systems, *Pf*Ago does not require a PAM sequence, and its guide DNA is relatively inexpensive and stable. The reaction temperature also helps suppress background signals [21,22]. MIRA-*Pf*Ago coupled assays have been used to detect several pathogens, demonstrating their potential for rapid nucleic acid testing [23,24]. A conserved region of the GAstV-2 *ORF1b* gene was selected to develop a MIRA-*Pf*Ago assay for GAstV detection. The assay was then assessed for sensitivity, specificity, and performance with clinical samples. This method may serve as a useful option for field diagnosis and epidemiological surveillance of GAstV-2 infection.

## 2. Materials and Methods

### 2.1. Viruses and Clinical Samples

Nucleic acids of GAstV-2, Tembusu virus (DTMUV), H9 subtype avian influenza virus (AIV-H9), goose circovirus (GoCV), fowl adenovirus serotype 4 (FAdV-4), and goose parvovirus (GPV) were available in our laboratory (Institute of Animal Husbandry and Veterinary Science, Anhui Academy of Agricultural Sciences, Hefei, China). The GAstV-1 nucleic acid sample was kindly provided by Professor Guijun Wang of Anhui Agricultural University. Twenty-three goose tissue samples suspected of GAstV-2 infection were collected from waterfowl farms in Anhui Province. The collection of clinical goose tissue samples and related animal procedures followed the regulations of the Animal Welfare and Ethics Committee of Anhui Academy of Agricultural Sciences. The protocol was reviewed and approved by this committee under approval number AAAS2025-1.

### 2.2. Nucleic Acid Extraction and Standard Plasmid Preparation

Clinical tissue samples were minced and placed in 1.5 mL centrifuge tubes. PBS was added at a 1:3 (w/v) ratio, and the samples were ground to form homogenates. After centrifugation at 12,000 rpm for 10 min at 4 °C, the supernatant was collected for nucleic acid extraction. Total nucleic acids were extracted using a viral DNA/RNA extraction kit (TIANGEN Biotech, Beijing, China) and stored at −80 °C until further use. For preparation of the standard plasmid, the target fragment of the *ORF1b* gene was cloned into the pUC57 vector to obtain pUC57-ORF1b. Plasmid copy number was calculated according to the total length of the recombinant plasmid, including both the pUC57 vector and the *ORF1b* insert, using the following equation:

Copy number per microlitre = concentration (ng/µL) × 6.022 × 10^23^/[3217 (bp) × 660 × 10^9^].

The quantified recombinant plasmid was diluted to the required concentration using nuclease-free water and stored at −20 °C until use.

### 2.3. Mira Primer Design and Validation

MIRA primers were designed according to the GAstV-2 *ORF1b* gene sequences deposited in GenBank (Table 1). Amplification was performed using an RNA rapid amplification kit (Weifang Amp-Future Biotech, Weifang, China) according to the manufacturer’s instructions. Each 25 μL reaction contained 14.7 μL of A Buffer, 1.0 μL of forward primer (10 μM), 1.0 μL of reverse primer (10 μM), 1.0 μL of nucleic-acid template, 1.25 μL of B Buffer, and ddH_2_O to the final volume. The commercial RNA MIRA reagent contains reverse-transcription activity; therefore, RNA-containing extracts were added directly to the RT-MIRA reaction without a separate cDNA-synthesis step. DNA templates were directly available for MIRA in the same reaction system. The reaction mixture was transferred to a tube containing lyophilized enzyme powder and incubated at 39 °C for 30 min. Amplification products were examined by agarose gel electrophoresis. The lyophilized enzyme formulation supplied with the kit contained reverse transcriptase; therefore, a separate reverse-transcription reaction was not required. Extracted viral RNA was added directly to the reaction, and reverse transcription and MIRA were performed simultaneously at 39 °C for 30 min. To assess the conservation and genotype specificity of the assay recognition regions, representative GAstV-2 *ORF1b* sequences and GAstV-1 *ORF1b* sequences were aligned. The binding sites of the MIRA primers, gDNA3, and molecular beacon were analysed, and the results are presented in Table S1 and Figure S1. 

### 2.4. gDNA Design and PfAgo Cleavage Validation

A 59 bp ssDNA corresponding to the MIRA target region was synthesized, and five 5′-phosphorylated guide DNAs (gDNAs) were designed for *Pf*Ago cleavage (Table 1). Each 20 μL cleavage reaction contained 2 μL of 10× Reaction Buffer (150 mM Tris/HCl, 1 M NaCl, pH 8.0), 0.2 μM *Pf*Ago (Jiaohong Biotech, Shanghai, China), 0.5 μM gDNA, 1 μM GAstV ssDNA, and 2.5 mM MnCl_2._ The reaction was incubated at 95 °C for 30 min, and the cleavage products were analyzed by 16% urea-denaturing PAGE. The gDNA showing the best cleavage performance was selected for subsequent assay development. A FAM-BHQ1-labeled molecular beacon was then designed based on the selected gDNA. All primers and probes were synthesized by General Biosystems (Anhui) Co., Ltd.

### 2.5. Establishment and Optimization of the RT-MIRA-PfAgo Assay

For fluorescence detection, 3 μL of MIRA product was added to a 20 μL PfAgo reaction system containing 1 μM gDNA3, 2.5 mM MnCl_2_, 0.6 μM PfAgo, 2 μL of 10× Reaction Buffer, 0.5 μM molecular beacon, and nuclease-free H_2_O. The reaction was carried out at 95 °C for 30 min. The PfAgo reaction was optimized by varying the concentration of gDNA3 (0, 0.5, 1, 1.5, and 2 μM), PfAgo (0, 0.2, 0.4, 0.6, and 0.8 μM), and MnCl_2_ (0.5, 1.0, 1.5, 2.0, 2.5, and 3.0 mM). Fluorescence was monitored on a Gentier 96R real-time PCR system (Tianlong Science and Technology, Xi’an, China). Reactions were performed at 95 °C for 30 min, with FAM signals collected every 30 s. The endpoint fluorescence threshold was defined as the mean RFU of the no-template controls plus three standard deviations. Tube images were acquired using a Tanon 4600 series fully automated chemiluminescence imaging system (Tanon Science & Technology Co., Ltd., Shanghai, China). All tubes within the same experiment were imaged simultaneously using the same imaging mode and acquisition parameters. To minimize amplicon carryover contamination, reagent preparation, template addition, amplification, and post-amplification analysis were performed using a unidirectional workflow. Pre-amplification and post-amplification procedures were conducted in separate working areas with dedicated pipettes and aerosol-resistant filter tips. Work surfaces and equipment were routinely treated with a nucleic-acid decontamination reagent and ultraviolet irradiation. 

### 2.6. Specificity and Sensitivity Assays

The specificity of the assay was evaluated using nucleic acids of DTMUV, AIV-H9, GoCV, FAdV-4, and GPV as templates. All non-target nucleic acid samples were confirmed to be positive using their respective pathogen-specific PCR or RT-PCR assays before cross-reactivity testing. The extracted nucleic acids were adjusted to concentrations of 20 ng/μL, and 1 μL of each nucleic acid sample was added to the 25 μL MIRA reaction. RNase-free ddH_2_O was included as the negative control. For sensitivity analysis, the recombinant plasmid pUC57-ORF1b was 10-fold serially diluted from 1.0 × 10^8^ to 1.0 × 10^0^ copies/μL. One microlitre of each dilution was added to the 25 μL MIRA reaction, corresponding to nominal template inputs ranging from 1.0 × 10^8^ to 1.0 × 10^0^ plasmid copies per reaction. The amplification products were then tested by *Pf*Ago-mediated cleavage. Fluorescence signals were recorded after the reaction to determine the lowest detectable plasmid concentration under the tested conditions.

### 2.7. Detection of Clinical Samples

The established RT-MIRA-*Pf*Ago assay was applied to 23 clinical tissue samples suspected of GAstV-2 infection. Conventional RT-PCR and RT-qPCR [25] were performed in parallel. RT-qPCR was used as the principal comparator method. Because no independent diagnostic gold standard was available, assay agreement was evaluated using positive percentage agreement, negative percentage agreement, overall agreement, and Cohen’s kappa coefficient. Exact 95% confidence intervals for proportions were calculated using the Clopper–Pearson method. The agreement analysis between the RT-MIRA-*Pf*Ago assay and RT-qPCR is summarised in Table S2, and the sample-level results obtained by RT-MIRA-*Pf*Ago, conventional RT-PCR, and RT-qPCR are provided in Table S3.

### 2.8. Data Processing and Statistical Analysis

All optimization, sensitivity, and specificity experiments were performed independently at least three times. Data are presented as the mean ± standard deviation from three independent experiments. Experiments involving more than two groups were analysed using one-way ANOVA followed by Tukey’s multiple-comparison test. Comparisons between two groups were performed using an unpaired two-tailed Student’s t-test. 

## 3. Results

### 3.1. Principle of the RT-MIRA-PfAgo Assay

**Figure 1:**
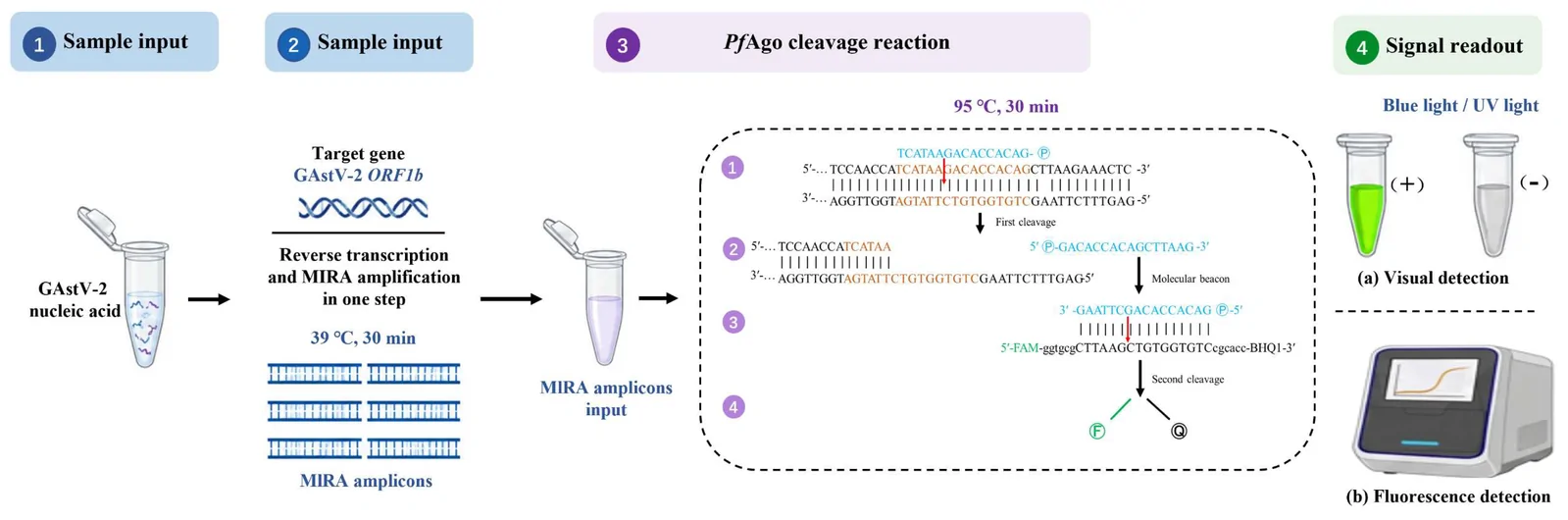
Schematic illustration of the RT-MIRA-*Pf*Ago assay for GAstV-2 detection.

The principle of the RT-MIRA-*Pf*Ago assay is shown in Figure 1. In samples containing GAstV-2 RNA, reverse transcription and MIRA are performed simultaneously to enrich the *ORF1b* target fragment. The amplification products are then used for *Pf*Ago-mediated cleavage at 95 °C, during which gDNA directs *Pf*Ago to recognize and cleave the target sequence. This initial cleavage generates secondary single-stranded DNA fragments, which further serve as guide DNAs for molecular beacon cleavage. After the molecular beacon is cleaved, the FAM fluorophore is separated from the BHQ1 quencher, producing a fluorescence signal. The released signal can be quantitatively measured using a fluorescence detector or qualitatively observed using a portable blue-light fluorescence visualization device. The assay requires constant-temperature incubation at 39 °C and 95 °C but does not require programmed thermal cycling or an expensive real-time PCR instrument for routine endpoint detection.

### 3.2. Validation of MIRA Primers

**Figure 2:**
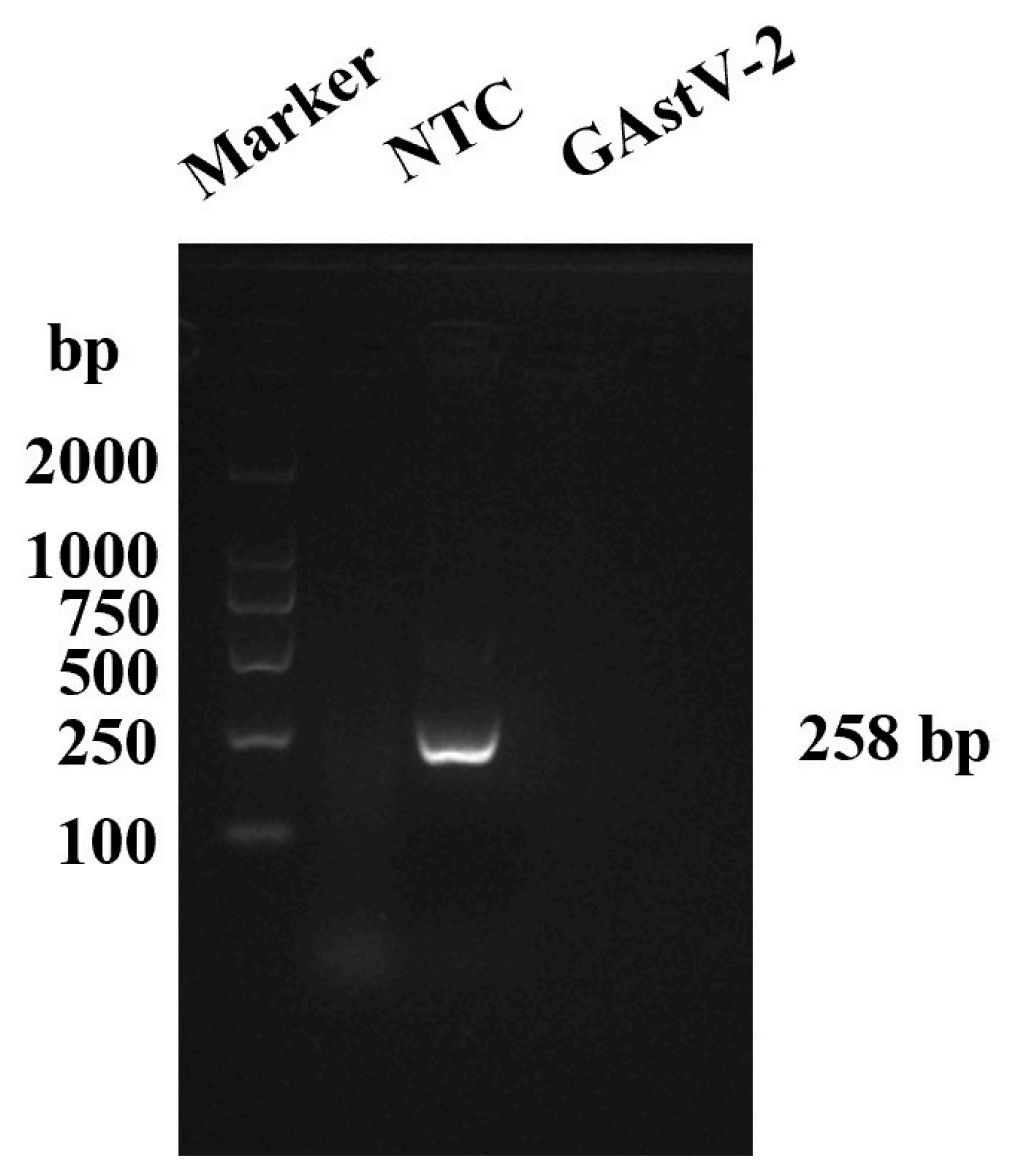
Gel electrophoresis of MIRA product. M, DNA molecular marker; NTC, no-template control; GAstV-2, MIRA reaction containing (pUC57-ORF1b plasmid).

The designed MIRA primers were tested using GAstV-2 nucleic acid as the template. After amplification, the products were examined by 2% agarose gel electrophoresis. A clear single band of approximately 258 bp was observed, matching the expected product size (Figure 2). This primer set was selected for the following experiments.

### 3.3. Screening of gDNA

**Figure 3:**
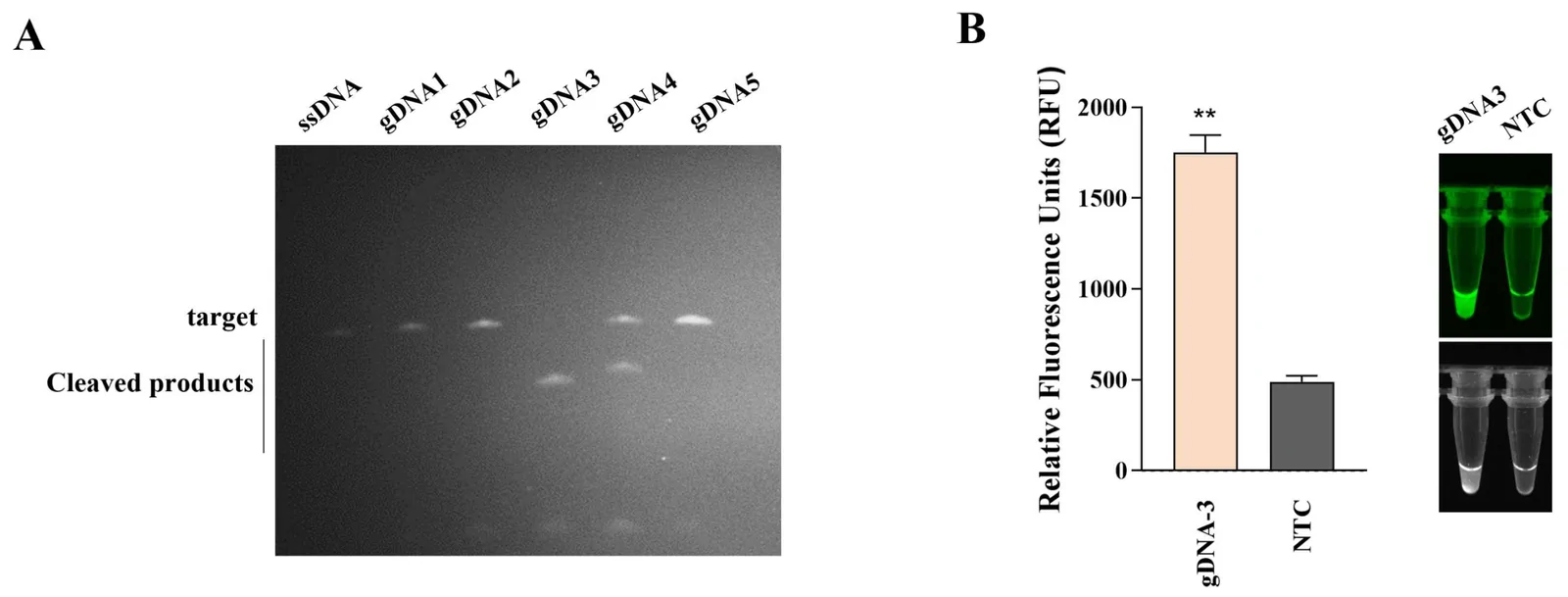
gDNA screening. (**A**) The screening result of gDNAs on 16% urea-denaturing PAGE. Lane ssDNA, uncleaved substrate control; lanes gDNA1–gDNA5, *Pf*Ago cleavage reactions guided by the corresponding gDNA. The upper band represents the intact substrate, whereas the lower bands represent cleavage products. (**B**) The detection fluorescence intensity and image results (visualized with a blue-light and UV) of the RT-MIRA-*Pf*Ago with the guidance of gDNA3. NTC: no-template control. ** *p*< 0.01.

gDNA guides *Pf*Ago to the target sequence, and different gDNAs may affect cleavage efficiency. To compare the five candidates on PAGE gels, a standard GAstV-2 ssDNA fragment corresponding to the *ORF1b* target region was used as the cleavage substrate. Among the five tested gDNAs, gDNA3 showed the most efficient cleavage, with nearly complete digestion of the target fragment (Figure 3A). gDNA3 was selected for subsequent assay construction.

The complete process, from MIRA to molecular beacon cleavage, was then examined using gDNA3. When GAstV-2 nucleic acid was used as the template, positive signals were detected by both fluorescence measurement and blue-light observation (Figure 3B). These results indicated that the RT-MIRA-*Pf*Ago system was feasible for the detection of GAstV-2 nucleic acids.

### 3.4. Optimization of the RT-MIRA-PfAgo Assay

**Figure 4:**
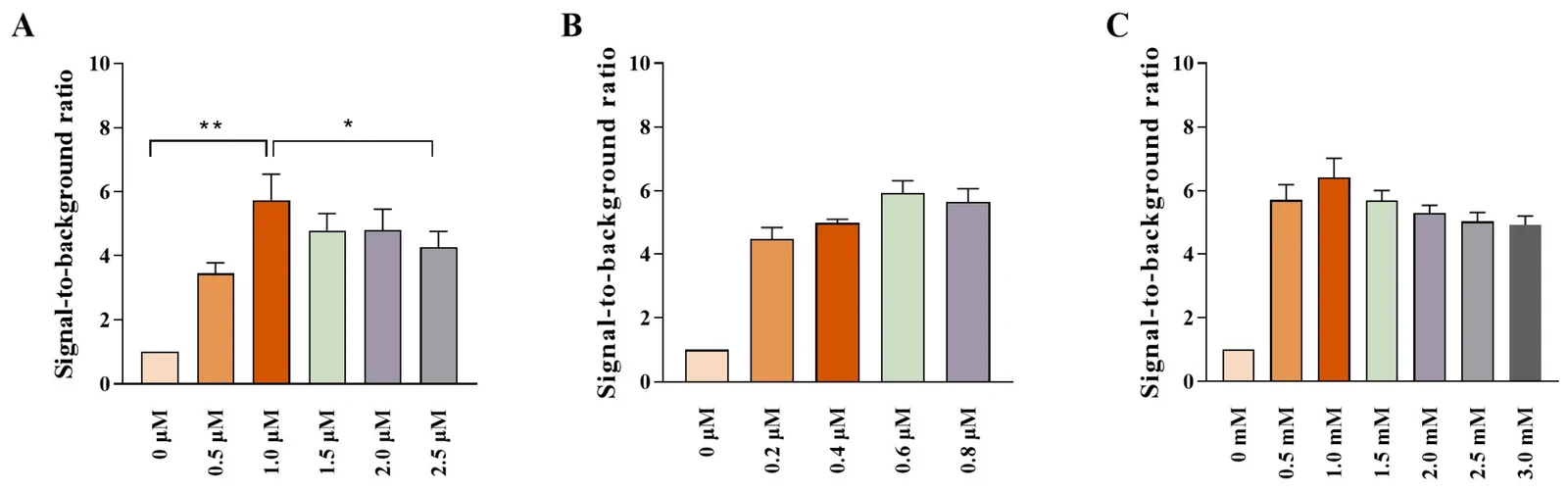
RT-MIRA-*Pf*Ago reaction optimization. Effects of gDNA concentration (**A**), *Pf*Ago concentration (**B**), and MnCl_2_ concentration (**C**) on the signal-to-background ratio. The signal-to-background ratio was calculated separately for each independent experimental run by dividing the endpoint RFU at each tested concentration by the RFU of the corresponding zero-component control. * *p* < 0.05, ** *p* < 0.01.

Key components of the *Pf*Ago reaction were adjusted to improve the fluorescence response, including the concentrations of gDNA, *Pf*Ago, and Mn^2+.^ The fluorescence intensity was highest at 1 μM gDNA (Figure 4A). For *Pf*Ago, the strongest signal was observed at 0.6 μM (Figure 4B). Among the tested MnCl_2_ concentrations, 1.0 mM produced the highest fluorescence peak (Figure 4C). Accordingly, 1.0 μM gDNA, 0.6 μM *Pf*Ago, and 1.0 mM MnCl_2_ were used as the reaction conditions in the following experiments.

### 3.5. Sensitivity and Specificity of the RT-MIRA-PfAgo Assays

**Figure 5:**
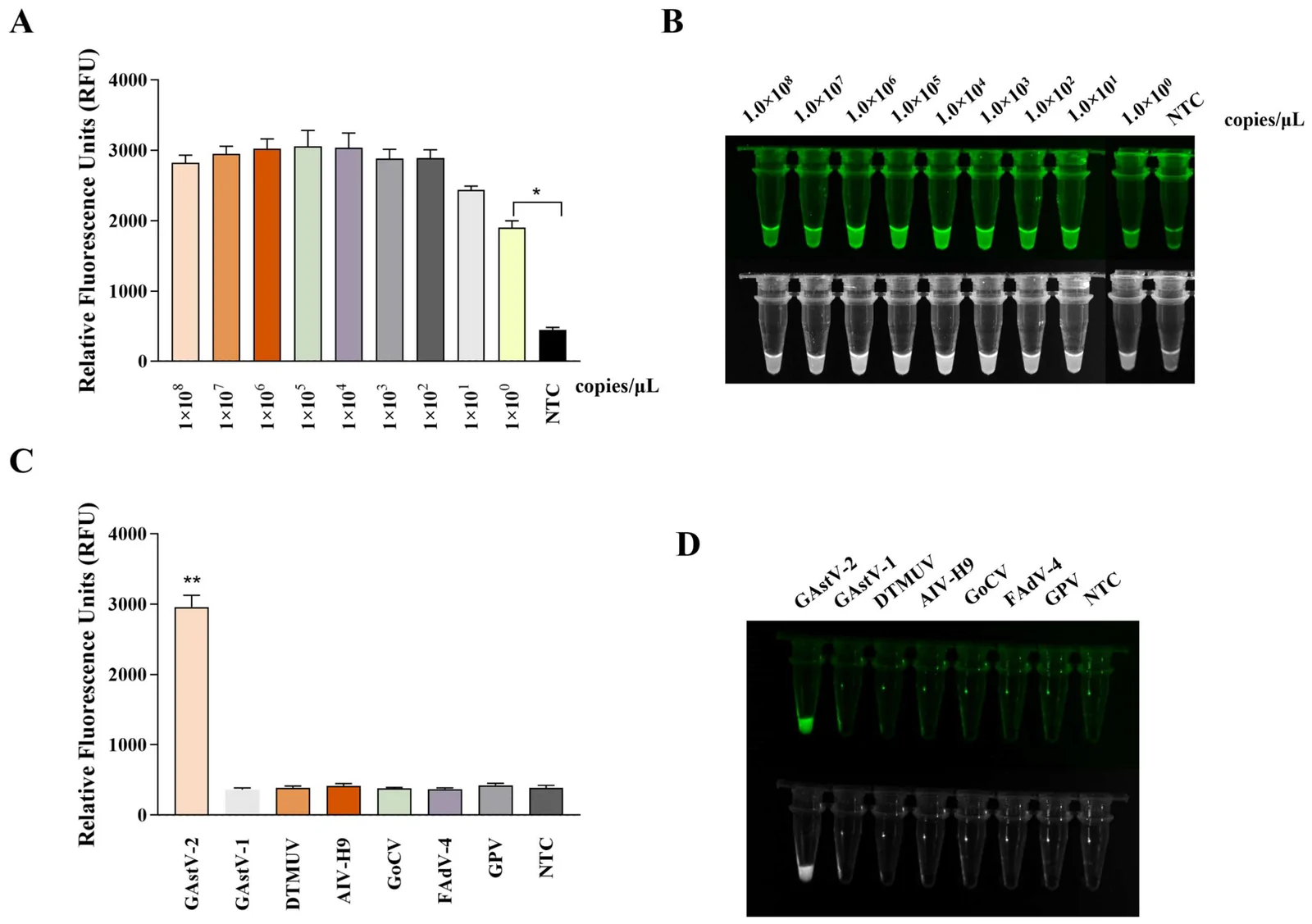
Sensitivity and specificity of the RT-MIRA-*Pf*Ago reaction. (**A**) Relative fluorescence units of RT-MIRA-*Pf*Ago sensitivity; (**B**) the images were captured under blue light; (**C**) relative fluorescence units of specificity testing; (**D**) the images were captured under UV light. The upper images were captured under blue light, and the lower images were captured under ultraviolet light. Error bars represent SEM; *n* = 3, * *p* < 0.05, ** *p* < 0.01.

The sensitivity of the assay was examined using 10-fold serial dilutions of the recombinant plasmid pUC57-ORF1b. Green fluorescence remained visible at 1.0 × 10^0^ copies/μL, and the RFU value at this concentration was significantly higher than that of the negative control (*P* < 0.05). Under these conditions, 1.0 × 10^0^ copies/μL was the lowest recombinant plasmid concentration producing a detectable fluorescence signal (Figure 5A, B).

Specificity was evaluated using nucleic acids from GAstV-2 and five common avian pathogens, including GAstV-1, DTMUV, AIV-H9, GoCV, FAdV-4, and GPV. A clear green fluorescence signal was observed only in the GAstV-2 reaction. No visible cross-reactive signal was observed in reactions containing the other tested pathogens (Figure 5C, D). Sequence-alignment analysis showed that the gDNA3, molecular-beacon, and MIRA-R recognition sites were completely conserved among the five analysed GAstV-2 sequences. The MIRA-F site was also highly conserved. In contrast, the GAstV-1 strains FLX and TZ03 contained multiple mismatches across all four recognition regions, supporting the intended GAstV-2-specific design of the assay (Table S1 and Figure S1).

### 3.6. Clinical Sample Detection Results

**Figure 6:**
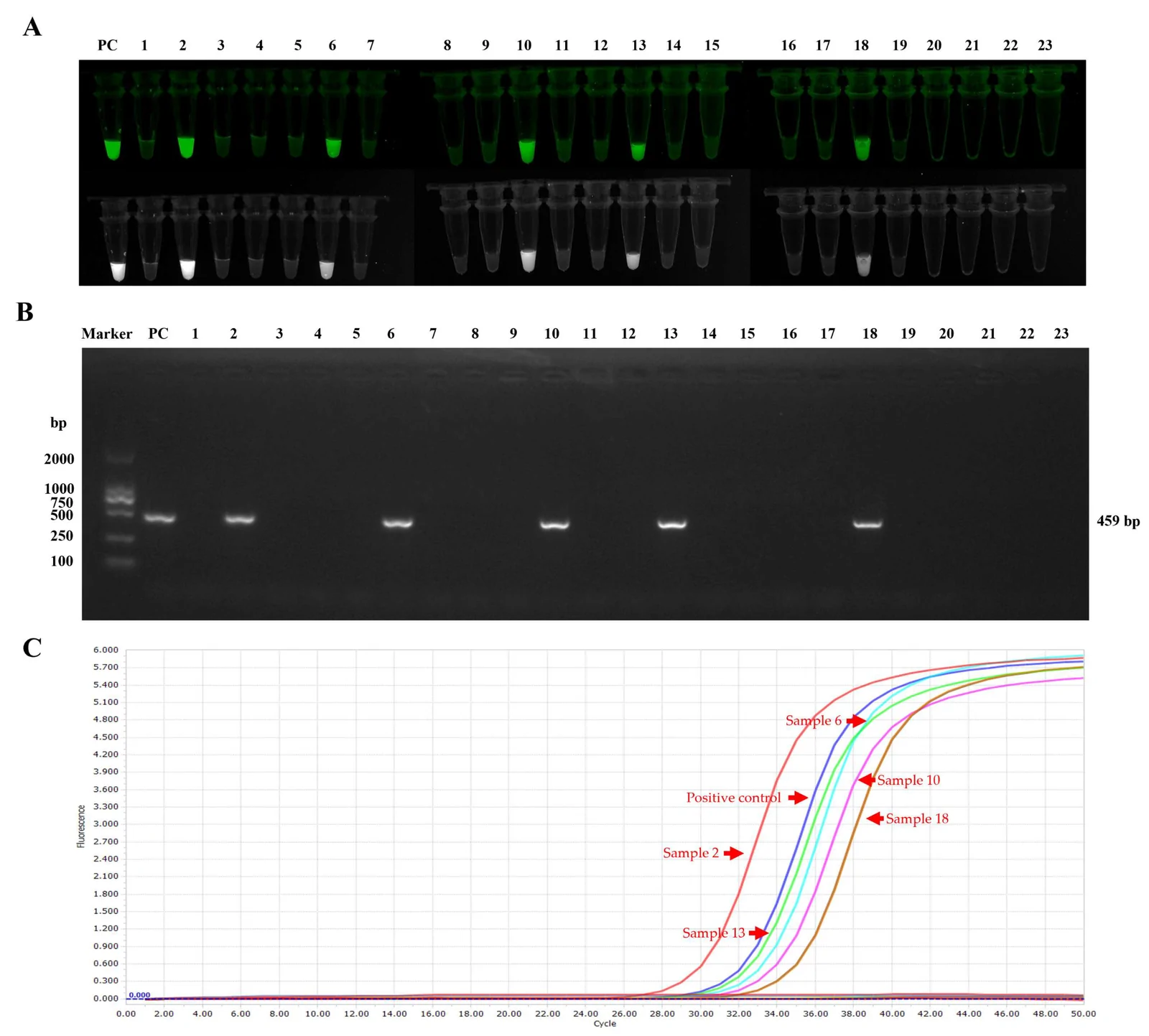
Comparative detection of 23 clinical goose tissue samples using RT-MIRA-*Pf*Ago, conventional RT-PCR, and RT-qPCR. (**A**) Endpoint RT-MIRA-*Pf*Ago results for the positive control and clinical samples 1–23. The upper images were captured under blue light, and the lower images were captured under ultraviolet light. (**B**) Agarose gel electrophoresis of conventional RT-PCR products. M, DNA molecular marker; PC, positive control; lanes 1–23, clinical samples 1–23, (**C**) RT-qPCR amplification curves obtained from the same clinical samples.

The established RT-MIRA-*Pf*Ago assay was applied to 23 clinical samples suspected of GAstV-2 infection, with conventional RT-PCR and RT-qPCR performed in parallel. Five samples tested positive by RT-MIRA-*Pf*Ago (Figure 6A), and the same five samples were also positive by conventional PCR and RT-qPCR (Figure 6B, C). Using RT-qPCR as the comparator, the positive percentage agreement was 100% (5/5; exact 95% CI, 47.8–100%), the negative percentage agreement was 100% (18/18; exact 95% CI, 81.5–100%), and the overall agreement was 100% (23/23; exact 95% CI, 85.2–100%). Cohen’s kappa coefficient was 1.00. The detailed agreement metrics are summarised in Table S2, and the individual results for all 23 clinical samples are listed in Table S3. These results demonstrate complete concordance within this limited sample set. In this sample set, the positive and negative results were consistent across the three methods. These findings support the applicability of the RT-MIRA-*Pf*Ago assay for detecting GAstV-2 in clinical tissue samples, although additional samples would be needed to evaluate its field performance.

## 4. Discussion

In recent years, GAstV-2, initially described as novel goose astrovirus, has become an important pathogen associated with visceral and articular gout in goslings [26,27]. The disease can spread rapidly within affected flocks and is often accompanied by high mortality, causing considerable economic losses to the waterfowl industry in China. Mortality rates of up to 50% have been reported in GAstV-2-infected goslings. Infections in ducks and chickens have also been described, indicating that the host range of GAstV-2 may extend beyond geese [8,9]. These features underscore the need for rapid, field-applicable detection methods for GAstV-2 control.

Several assays have been reported for GAstV-2 detection, including RT-PCR, RT-qPCR, LAMP, and CRISPR/Cas-based methods [3,18,28]. Yin et al. developed a TaqMan one-step RT-qPCR assay with a detection limit of 10 copies/μL [12]. The assay showed good sensitivity and repeatability, and its clinical positive detection rate was higher than that of conventional RT-PCR. However, it still requires a real-time PCR instrument, and the entire testing process usually takes 1.5–2 h, limiting its use for rapid detection at the farm level. Yang et al. established a duplex RT-qPCR assay for simultaneous detection of GAstV-2 and goose parvovirus, with a detection limit of 6.58 × 10^1^ copies/μL for GAstV-2 [25]. This method also depends on relatively expensive thermal cycling equipment. In the present study, the RT-MIRA-*Pf*Ago assay reached a detection limit of 1.0 × 10^0^ copies/μL. Under the tested conditions, its detection limit was lower than that reported for some qPCR assays, and the assay could be completed within about 60 min without a conventional thermal cycler.

Isothermal amplification methods have received increasing attention because they reduce the need for thermal cycling instruments. He et al. reported an RT-LAMP assay for GAstV-2 detection, which completed amplification at 65 °C within 60 min and had a detection limit of approximately 10^1^ copies/μL [29]. This method has potential for field use. However, LAMP usually requires four to six primers, and primer dimers or nonspecific amplification may increase the risk of false-positive results. The MIRA reaction used here requires only one pair of primers and amplifies the target at 39 °C. Because reverse transcriptase was included in the commercial RNA MIRA reagent, no separate cDNA synthesis step was required, thereby simplifying the assay workflow. The addition of *Pf*Ago-mediated cleavage provides a second sequence-recognition step after amplification, thereby improving the specificity of signal generation.

CRISPR/Cas systems have also been introduced into isothermal nucleic acid detection to improve specificity. Yang et al. established an RT-ERA-Cas12a assay for GAstV-2 detection at 37 °C, with fluorescence-based visual readout and a detection limit of 2 × 10^0^ copies [28]. This assay showed good sensitivity. However, Cas12a-based detection is dependent on the availability of an appropriate PAM sequence, which may restrict the selection of target sites. In contrast, *Pf*Ago is a DNA-guided endonuclease that uses short 5′-phosphorylated gDNAs and does not require a PAM sequence, allowing greater flexibility in selecting cleavage sites within the conserved GAstV-2 *ORF1b* region. The DNA guides used by *Pf*Ago are also relatively stable, easy to synthesize and store, and less susceptible to degradation than RNA guides. In addition, the high-temperature *Pf*Ago reaction promotes denaturation of the double-stranded MIRA products and facilitates guide–target recognition. Importantly, primary *Pf*Ago cleavage generates a 5′-phosphorylated DNA fragment that serves as a secondary guide for cleavage of the FAM/BHQ1-labelled molecular beacon. This sequential guide-dependent cleavage provides an additional sequence-recognition step after MIRA and is particularly suitable for the fluorescence signal-generation strategy used in this study. Although no direct comparison with a CRISPR/Cas assay was performed, these characteristics support the selection of *Pf*Ago as an appropriate detection component for the present assay. Nevertheless, the requirement for a 95 °C cleavage step remains a limitation of the current system.

Current rapid nucleic-acid detection strategies are progressing from stand-alone amplification toward integrated amplification, sequence-specific recognition, and portable signal readout. Recombinase-based methods such as RPA, RAA, ERA, and MIRA reduce the requirement for programmed thermal cycling, whereas CRISPR/Cas and prokaryotic Argonaute systems provide an additional sequence-recognition layer after amplification. Lateral-flow strips, colorimetric indicators, portable fluorescence devices, and microfluidic platforms can further simplify endpoint interpretation and support decentralized testing. Nevertheless, the practical performance of these systems depends not only on analytical sensitivity but also on reaction integration, contamination control, equipment requirements, reagent stability, and ease of operation.

The principal advantage of the present RT-MIRA-*Pf*Ago assay is the combination of low-temperature target amplification with a second sequence-specific *Pf*Ago cleavage step and portable fluorescence visualization. The assay therefore provides greater sequence discrimination than MIRA used alone and does not require a conventional thermal cycler or a real-time PCR instrument for routine endpoint detection. However, the current workflow requires sequential incubations at 39 °C and 95 °C and transfer of the RT-MIRA product into the *Pf*Ago reaction tube. Consequently, it is less integrated than a closed-tube or one-pot point-of-care platform and remains susceptible to amplicon carryover contamination. Development of a sealed single-tube format will therefore be an important priority for future optimization.

To better compare the present assay with reported GAstV-2 detection methods, their detection limits, detection times, equipment requirements, and field-use potential are summarized in Table 2. Compared with these methods, the RT-MIRA-*Pf*Ago assay combined a low detection limit, sequence-specific recognition, and visual readout. It also reduced dependence on precision instruments, supporting its potential for field-oriented testing.

Although the RT-MIRA-*Pf*Ago assay requires two constant-temperature incubation steps, at 39 °C and 95 °C, both steps can be performed using a simple thermostatic metal bath or dry-block heater. The real-time PCR system used in this study was employed only for quantitative fluorescence monitoring during assay optimization. Routine endpoint results can be observed using a portable blue-light fluorescence visualization device. Therefore, the assay reduces dependence on conventional thermal cyclers and laboratory fluorescence instruments, although basic temperature-control and fluorescence-observation equipment remain necessary.

In the specificity assay, no cross-reaction was observed with nucleic acids from DTMUV, AIV-H9, GPV, GoCV, or FAdV-4. This result indicates that the assay specifically detected GAstV-2 under the tested conditions. In the clinical evaluation, 23 suspected tissue samples were tested by RT-MIRA-*Pf*Ago, conventional PCR, and RT-qPCR. The same five samples were positive by all three methods, and the positive and negative results were consistent in this sample set. These data support the potential use of the RT-MIRA-*Pf*Ago assay for detecting GAstV-2 in clinical tissue samples. The fluorescence signal could also be observed under blue or ultraviolet light after the reaction, reducing the need for fluorescence detection equipment.

This study still has several limitations. The current assay uses a two-step format in which the RT-MIRA amplification product must be transferred to the *Pf*Ago cleavage reaction. Although unidirectional operation, spatial separation of pre- and post-amplification procedures, aerosol-resistant filter tips, surface decontamination, and no-template controls were used to reduce contamination during the present study, a formal carryover-contamination challenge was not performed. Therefore, the risk of aerosol contamination under routine laboratory or field conditions remains to be determined. Future work should focus on developing a sealed single-tube or physically compartmentalized format. Potential strategies include separating the RT-MIRA and *Pf*Ago reagents using a wax or phase-change barrier, storing the *Pf*Ago components in the tube cap, or using sealed reaction compartments that mix only after amplification. Such formats could permit sequential reactions at 39 °C and 95 °C without opening the tube, thereby reducing amplicon exposure and improving operational safety.

## 5. Conclusions

A visual rapid assay combining MIRA with *Pf*Ago-mediated cleavage was developed for GAstV-2 detection. The assay targets a conserved region of the GAstV-2 *ORF1b* gene and integrates isothermal amplification with sequence-specific nucleic acid cleavage, allowing detection within about 60 min. Using recombinant plasmid DNA, the lowest detectable concentration under the tested conditions was 1.0 × 10^0^ copies/μL. No cross-reaction was observed with common waterfowl pathogens, and the clinical results were consistent with those obtained by conventional PCR and RT-qPCR. The RT-MIRA-*Pf*Ago assay showed favorable analytical sensitivity and specificity under the tested conditions and enabled both fluorescence-based and visual endpoint detection. These features make it a practical option for basic laboratories and on-site testing. The assay may also support rapid diagnosis and epidemiological surveillance of GAstV-2 infection, although further validation with larger clinical sample sets is still needed. 

## Figures and Tables

**Table 1:** Sequences used in this study.

Reaction	Name	Sequence (5′-3′)
PCR	GAstV-F	TCATTACGAGCAGGAGAA
	GAstV-R	CTGCACCGCAACTTTAAA
MIRA	MIRA-F	TCTGGCGGATACGACAGATGCGTTACTT
	MIRA-R	TCGGCAATGACCCATGCTGTTTCCAATA
	GAstV-ssDNA	TCCAACCATCATAAGACACCACAGCTTAAGAAACTCT ATGATTGGTATGTTAAAAACCT
*Pf*Ago	gDNA1	P-TCTTATGATGGTTGGA
	gDNA2	P-GGTGTCTTATGATGGT
	gDNA3	P-GACACCACAGAATACT
	gDNA4	P-TAAGCTGTGGTGTCTT
	gDNA5	P-TTCTTAAGCTGTGGTG
	MB	FAM-ggtgcgCTTAAGCTGTGGTGTCcgcacc-BHQ1

Note: GAstV refers to GAstV-2 in the assay oligonucleotide names.

**Table 2:** Comparison of existing Detection Methods for Goose Astrovirus.

Method	Material Used for Sensitivity Assessment	Reported Analytical Threshold	Detection Time	Liquid-Transfer Steps After Template Addition	Tube Format	Readout Method	Minimum Required Instruments
TaqMan one-step RT-qPCR [10]	Quantified RNA standard	10 copies/μL	1.5–2 h	0	Closed tube	Real-time TaqMan fluorescence	Real-time PCR instrument
Duplex SYBR Green I qPCR [25]	DNA standards; GAstV clinical testing used separately prepared cDNA	6.58 × 10^1^ copies/μL	1.5–2 h	1	Closed during qPCR	SYBR Green fluorescence and melt-curve discrimination	Real-time PCR instrument
qLAMP [29]	pMD18-ORF1a recombinant plasmid DNA	1 × 10^1^ copies/μL	60 min	1	Closed for real-time or endpoint fluorescence; opened for gel confirmation	EvaGreen fluorescence, UV/blue-light endpoint observation, or agarose gel electrophoresis	65 °C water bath or heat block and UV/blue-light viewer; real-time PCR instrument optional
RT-ERA–Cas12a [28]	pMD-19T-ORF2 recombinant plasmid DNA	2×10^0^ copies/μL	60 min	1	Open, two-step	Fluorophore–quencher reporter; LED blue light, UV, or real-time fluorescence	Constant-temperature heater/water bath and blue-light or UV viewer
RT-MIRA–*Pf*Ago (this study)	pUC57-ORF1b recombinant plasmid DNA	10^0^ copy/μL; 1 μL input, 1 copy/reaction	60 min	1	Open, two-step	FAM/BHQ1 fluorescence; portable blue-light or UV endpoint observation; real-time fluorescence optional for optimization	Metal bath or dry-block heater and portable fluorescence viewer

## Data Availability

The data presented in this study are available in the article. Additional raw data supporting the findings of this study are available from the corresponding author upon reasonable request.

## Supplementary Materials

The following supporting information can be downloaded at: https://www.mdpi.com/article/10.3390/bios16080433/s1, Figure S1: Multiple-sequence alignment of the RT-MIRA-PfAgo recognition sites in representative GAstV-1 and GAstV-2 ORF1b sequences. The MIRA-F binding site (A), gDNA3 target-recognition site (B), molecular-beacon target site (C), and MIRA-R binding site (D) were aligned against the assay target sequences. Dots indicate identity, letters indicate nucleotide substitutions, and dashes indicate alignment gaps. Percentages indicate site-specific nucleotide identity. The analysed GAstV-2 sequences were completely conserved at the gDNA3, molecular-beacon, and MIRA-R sites; JSHA contained one internal mismatch in the MIRA-F site; Table S1: Conservation of the RT-MIRA-PfAgo recognition sites among representative novel goose astrovirus (GAstV-2) strains and sequence discrimination from GAstV-1 strains. Cati. A. Representative sequences included in the analysis. B. Site-level conservation and mismatch assessment; Table S2: Agreement between the RT-MIRA-PfAgo assay and RT-qPCR for the detection of GAstV-2 in clinical samples (*n* = 23); Table S3: Sample-level comparison of RT-MIRA-PfAgo, conventional RT-PCR, and RT-qPCR results for GAstV-2 detection in clinical goose tissue samples.

## Abbreviations

The following abbreviations are used in this manuscript:

qPCR	quantitative real-time PCR
MIRA	multienzyme isothermal rapid amplification
PAM	protospacer adjacent motif
NTC	no-template control
PC	positive control
