# Supplementary material for: Development of a Visual Rapid Assay for Novel Goose Astrovirus Detection Based on RT-MIRA -PfAgo
Source: Biosensors (Basel). 2026 Aug 8;16(8):433. doi: 10.3390/bios16080433 (PMC13511664; doi:10.3390/bios16080433)
Supplement: Supplementary file 1 [file biosensors-16-00433-s001.zip › biosensors-4444100-supplementary.pdf]

biosensors

Communication

Development of a Visual Rapid Assay for Novel Goose Astrovirus Detection Based on RT-MIRA -*Pf*Ago

Dongdong Yin [1,*], Xinjun Chen [2], Zhixing Cheng [2], Yu Liu [2], Yin Dai [1], Xuehuai Shen [1] and Xiaocheng Pan [1,*]

1 Anhui Provincial Key Laboratory of Livestock and Poultry Product Safety, Institute of Animal Husbandry and Veterinary Science, Anhui Academy of Agricultural Sciences, Livestock and Poultry Epidemic Diseases Research Center of Anhui Province, Hefei 230031, China; dalin2080@aaas.org.cn (Y.D.); shenxh@aaas.org.cn (X.S.)

2 Yebio Bioengineering Co., Ltd of Qingdao, Qingdao 266113, China; chenxinjun@yebio.com (X.C.); chengzhixing@yebio.com (Z.C.); liuyu344@yebio.com (Y.L.)

* Correspondence: ydd@aaas.org.cn (D.Y.); xiaochengpan@aaas.org.cn (X.P.)

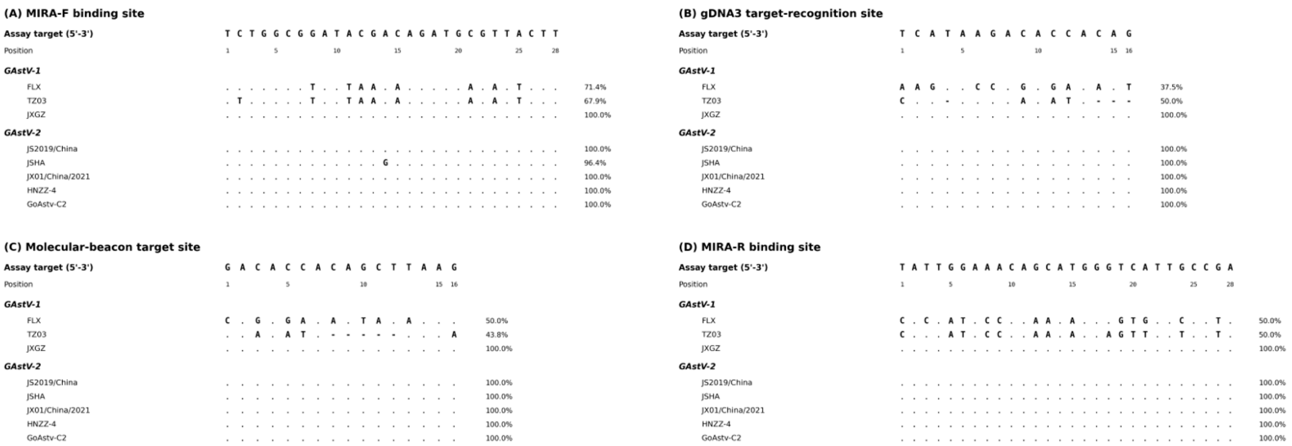

Figure S1. Multiple-sequence alignment of the RT-MIRA-PfAgo recognition sites in representative GAstV-1 and GAstV-2 ORF1b sequences. The MIRA-F binding site (A), gDNA3 target-recognition site (B), molecular-beacon target site (C), and MIRA-R binding site (D) were aligned against the assay target sequences. Dots indicate identity, letters indicate nucleotide substitutions, and dashes indicate alignment gaps. Percentages indicate site-specific nucleotide identity. The analysed GAstV-2 sequences were completely conserved at the gDNA3, molecular-beacon, and MIRA-R sites; JSHA contained one internal mismatch in the MIRA-F site.

 https://doi.org/10.3390/bios16080433

Table S1. Conservation of the RT-MIRA-*Pf*Ago recognition sites among representative novel goose astrovirus (GAstV-2) strains and sequence discrimination from GAstV-1 strains. cati.

A. Representative sequences included in the analysisR pe.

Genotype	Strain	GenBank accession	Geographic origin	Year
GAstV-1	FLX	NC_034567.1	Hunan, China	2014
GAstV-1	TZ03	MW353015.1	Jiangsu, China	2019
GAstV-1	JXGZ	OL762471.1	Jiangxi, China	2021
GAstV-2	JS2019/China	MZ540211.1	Jiangsu, China	2019
GAstV-2	JSHA	MK125058.1	Jiangsu, China	2018
GAstV-2	JX01/China/2021	MZ576222.1	Jiangxi, China	2021
GAstV-2	HNZZ-4	ON376722.1	Henan, China	2021
GAstV-2	GoAstv-C2	OP179660.1	Sichuan, China	2022

B. Site-level conservation and mismatch assessment.

Recognition element	GAstV-1 exact match n/N; identity	GAstV-2 exact match n/N; identity	Overall exact match n/N; identity
MIRA-F	1/3 (33.3%); 79.8%	4/5 (80.0%); 99.3%	5/8 (62.5%); 92.0%
gDNA3 target	1/3 (33.3%); 62.5%	5/5 (100.0%); 100.0%	6/8 (75.0%); 85.9%
Molecular-beacon target	1/3 (33.3%); 64.6%	5/5 (100.0%); 100.0%	6/8 (75.0%); 86.7%
MIRA-R target	1/3 (33.3%); 66.7%	5/5 (100.0%); 100.0%	6/8 (75.0%); 87.5%

Table S2. Agreement between the RT-MIRA-*Pf*Ago assay and RT-qPCR for the detection of GAstV-2 in clinical samples (n = 23).

MIRA-*Pf*Ago Assay	RT-qPCR		Total
	Positive	Negative	
Positive	5	0	5
Negative	0	18	18
Total	5	18	23

Diagnostic Metric	Formula	Value (%)	95% Confidence Interval (%) *
Sensitivity	TP / (TP + FN)	100.0%	47.8% – 100.0%
Specificity	TN / (TN + FP)	100.0%	81.5% – 100.0%
Positive Predictive Value (PPV)	TP / (TP + FP)	100.0%	47.8% – 100.0%
Negative Predictive Value (NPV)	TN / (TN + FN)	100.0%	81.5% – 100.0%
Overall Agreement (Accuracy)	(TP + TN) / Total	100.0%	85.2% – 100.0%

Table S3. Sample-level comparison of RT-MIRA-*Pf*Ago, conventional RT-PCR, and RT-qPCR results for GAstV-2 detection in clinical goose tissue samples.

Sample ID	Sample type	RT-MIRA-*Pf*Ago result	Conventional RT-PCR result	RT-qPCR result	RT-qPCR Ct value
1	Kidney	Negative	Negative	Negative	Undetermined
2	Kidney	Positive	Positive	Positive	28.60
3	Kidney	Negative	Negative	Negative	Undetermined
4	Liver	Negative	Negative	Negative	Undetermined
5	Liver	Negative	Negative	Negative	Undetermined
6	Kidney	Positive	Positive	Positive	31.15
7	Kidney	Negative	Negative	Negative	Undetermined
8	Liver	Negative	Negative	Negative	Undetermined
9	Kidney	Negative	Negative	Negative	Undetermined
10	Kidney	Positive	Positive	Positive	32.49
11	Liver	Negative	Negative	Negative	Undetermined
12	Kidney	Negative	Negative	Negative	Undetermined
13	Kidney	Positive	Positive	Positive	30.79
14	Liver	Negative	Negative	Negative	Undetermined
15	Kidney	Negative	Negative	Negative	Undetermined
16	Kidney	Negative	Negative	Negative	Undetermined
17	Kidney	Negative	Negative	Negative	Undetermined
18	Kidney	Positive	Positive	Positive	33.52
19	Liver	Negative	Negative	Negative	Undetermined
20	Kidney	Negative	Negative	Negative	Undetermined
21	Liver	Negative	Negative	Negative	Undetermined
22	Liver	Negative	Negative	Negative	Undetermined
23	Kidney	Negative	Negative	Negative	Undetermined
Summary	23 samples	5 positive; 18 negative	5 positive; 18 negative	5 positive; 18 negative	—

Notes: RT-qPCR was used as the principal comparator method. Based on the present sample set, positive percentage agreement was 100% (5/5; exact 95% CI, 47.8–100%), negative percentage agreement was 100% (18/18; exact 95% CI, 81.5–100%), overall agreement was 100% (23/23; exact 95% CI, 85.2–100%), and Cohen's kappa was 1.00. These estimates should be interpreted cautiously because only five positive samples were included.

Abbreviations:

Ct, cycle threshold;
CI, confidence interval;
NA, not available.

https://doi.org/10.3390/bios16080433
